# Identification of miR-1 and miR-499 in chronic atrial fibrillation by bioinformatics analysis and experimental validation

**DOI:** 10.3389/fcvm.2024.1400643

**Published:** 2024-08-16

**Authors:** Xinpei Chen, Yu Zhang, He Meng, Guiying Chen, Yongjiang Ma, Jian Li, Saizhe Liu, Zhuo Liang, Yinuo Xie, Ying Liu, Hongyang Guo, Yutang Wang, Zhaoliang Shan

**Affiliations:** ^1^ Munich Medical Research School, Ludwig-Maximilians University Munich, Munich, Germany; ^2^ Department of Cardiology, Chinese PLA General Hospital, Beijing, China; ^3^ Department of Cardiac Arrhythmia, Fuwai Yunnan Hospital, Chinese Academy of Medical Sciences, Kunming Medical University, Kunming, Yunnan, China; ^4^ Department of Cardiology, Beijing Anzhen Hospital, Beijing, China; ^5^ Department of Cardiology, Tianjin Chest Hospital, Tianjin, China; ^6^ Department of Pneumology, Tianjin Chest Hospital, Tianjin, China; ^7^ Department of Cardiology, The Sixth Medical Center of PLA General Hospital, Beijing, China; ^8^ Department of Cardiology, Beijing Jing Mei Group General Hospital, Beijing, China; ^9^ Department of Geriatric Cardiology, Chinese PLA General Hospital, Beijing, China

**Keywords:** atrial fibrillation, differentially expressed genes, bioinformatics, microRNAs (miRNAs), miRNA-mRNA regulatory network

## Abstract

**Background:**

Atrial fibrillation (AF) is one of the most prevalent arrhythmias and is characterized by a high risk of heart failure and embolic stroke, yet its underlying mechanism is unclear. The primary goal of this study was to establish a miRNA–mRNA network and identify the miRNAs associated with chronic AF by bioinformatics and experimental validation.

**Methods:**

The GSE79768 dataset was collected from the Gene Expression Omnibus(GEO) database to extract data from patients with or without persistent AF. Differentially expressed genes (DEGs) were identified in left atrial appendages (LAAs). The STRING platform was utilized for protein–protein interaction (PPI) network analysis. The target miRNAs for the top 20 hub genes were predicted by using the miRTarBase Web tool. The miRNA–mRNA network was established and visualized using Cytoscape software. The key miRNAs selected for verification in the animal experiment were confirmed by miRwalk Web tool. We used a classic animal model of rapid ventricular pacing for chronic AF. Two groups of animals were included in the experiment, namely, the ventricular pacing group (VP group), where ventricular pacing was maintained at 240–280 bpm for 2 weeks, and the control group was the sham-operated group (SO group). Finally, we performed reverse transcription-quantitative polymerase chain reaction (RT–qPCR) to validate the expression of miR-1 and miR-499 in LAA tissues of the VP group and the SO group. Left atrial fibrosis and apoptosis were evaluated by Masson staining and caspase-3 activity assays, respectively.

**Results:**

The networks showed 48 miRNAs in LAA tissues. MiR-1 and miR-499 were validated using an animal model of chronic AF. The expression level of miR-1 was increased, and miR-499 was decreased in VP group tissues compared to SO group tissues in LAAs (*P* < 0.05), which were correlated with left atrial fibrosis and apoptosis in AF.

**Conclusion:**

This study provides a better understanding of the alterations in miRNA-1 and miR-499 in chronic AF from the perspective of the miRNA–mRNA network and corroborates findings through experimental validation. These findings may offer novel potential therapeutic targets for AF in the future.

## Introduction

The incidence and prevalence of atrial fibrillation (AF) are rapidly increasing worldwide. According to data from the Framingham Heart Study, the prevalence of AF has tripled over the last five decades. The Global Burden of Disease project estimated the prevalence of AF to be approximately 46.3 million people worldwide in 2016 (1, 2). AF can severely impact patients' quality of life due to its association with serious complications, including stroke, heart failure, cognitive impairment, and sudden cardiac arrest, which result in increased morbidity, mortality, and healthcare costs (3, 4).

AF is generally considered to be a progressive condition, with patients typically progressing from paroxysmal to persistent and then long-standing persistent (chronic or permanent) forms. However, not all patients go through each phase, and the duration of each phase can vary widely. Decades of research have identified numerous pathophysiological processes contributing to the initiation, maintenance, and progression of AF (5). The underlying mechanisms of wear-and-tear, congenital, and genetic AF have been only partially elucidated. Electrical and structural remodeling of the atrial tissue are believed to contribute to the development of AF, with fibrosis playing an important role in this process (6). Atrial fibrosis has been shown to be an important part of the arrhythmogenic substrate, with an essential function in generating conduction abnormalities that contribute to AF perpetuation (7). Paroxysmal AF is more commonly associated with ectopic activities originating from the pulmonary veins (8).

MicroRNAs (miRNAs) are small noncoding RNAs that regulate gene expression post-transcriptionally. These molecules are vital in cellular activities, including cell growth, differentiation, development, and apoptosis (9). Abnormal levels of miRNAs in cardiac cells or even in the blood can promote AF via atrial remodeling (10, 11) data demonstrating a role for miRNAs in the pathophysiology of AF. MiRNAs have been investigated for many years as potential non-invasive biomarkers of several diseases and, as a future direction, possible therapeutic approaches for AF treatments (12). Multiple miRNAs are involved in controlling AF, and AF of different sorts has distinct underlying mechanisms, and different miRNAs may be involved in different types of AF (13). MiRNA mimics and inhibitors currently in preclinical development show promise as novel therapeutic agents for various diseases (14).

In this study, we utilized the GSE79768 (AF mRNA dataset), which contains expression profiles of patients with persistent AF and sinus rhythm, to identify potential key genes. We performed protein-protein interaction (PPI) analysis and the miRNA–mRNA network analysis and validated two miRNAs in animal AF models. Our study is expected to provide new insights into the pathogenesis and mechanisms of AF through both bioinformatics analyses and experimental verification.

## Methods

### Data acquisition

We retrieved gene expression profiles from the Gene Expression Omnibus(GEO) database, which is affiliated with the National Center for Biotechnology Information (NCBI) (https://www.ncbi.nlm.nih.gov). The keywords “persistent atrial fibrillation,” “Homo sapiens,” “left atrium,” and “expression profiling by the microarray” were used for the search. After screening and principal component analysis (PCA) plots checking, we selected the GSE79768 (AF mRNA dataset) to obtain gene expression profiles. This dataset is AF patients who underwent valvular surgery. 13 patients with persistent AF had a known AF duration of more than six months, while 6 patients with sinus rhythm (SR) did not have clinical evidence of AF and were not using any anti-arrhythmic drugs. All specimens were included in the analysis.

### Gene expression analysis

We compared the gene expression between the left atrial appendages (LAAs) of patients with persistent AF and those with SR.

### Identification of differentially expressed genes

Differentially expressed genes (DEGs) were identified using the LIMMA package [version 3.52.2] (15, 16) and visualized using ggplot2 [version 3.3.6] and ComplexHeatmap [version 2.13.1] (17) in R [version 4.2.1]. The data were normalized again using the normalizeBetweenArrays function of the LIMMA package. Probes corresponding to multiple molecules were removed, and for probes corresponding to the same molecule, only the probe with the maximum signal value was retained. Sample clustering between groups was assessed using PCA plots. Differential expression analysis between the two groups was performed using the LIMMA package, and the results were visualized using a volcano plot. The significantly expressed molecules were also visualized using a heatmap. DEGs were defined using thresholds of |logFC|>1 and adjusted *p*-value < 0.05, where FC represents fold change.

### Protein–protein interaction network analysis

To construct a PPI network based on the identified DEGs, we used the Search Tool for the Retrieval of Interacting Genes (STRING) database [version 11.5] (https://string-db.org/). The cytoHubba plugin of Cytoscape was employed to identify key genes, also known as hub genes, in the network using the maximal clique centrality (MCC) method. The top 20 hub genes were identified.

### Prediction of target miRNAs

We predicted target miRNAs for the top 20 hub genes using the miRTarBase Web tool (18), updated to 2022. Only miRNAs with strong evidence were selected. The miRNA–mRNA network was established by combining mRNA–miRNA interactions, and the key miRNAs selected for verification in the animal experiment were also confirmed by miRwalk Web tool version 3 (19). The regulatory network was visualized using Cytoscape software.

### Animals

All experiments involving canines were conducted following the approved protocols by the Institutional Animal Care and Use Committee of the Chinese PLA General Hospital. The study adheres to the ARRIVE 2.0 guidelines for reporting research involving animals. Throughout the study, all methods were in strict accordance with the relevant institutional guidelines and regulations. Every effort was made to minimize animal suffering and to minimize the number of experimental subjects required to obtain accurate and reliable data.

### Experimental model for atrial fibrillation in canines

**Figure 4:**
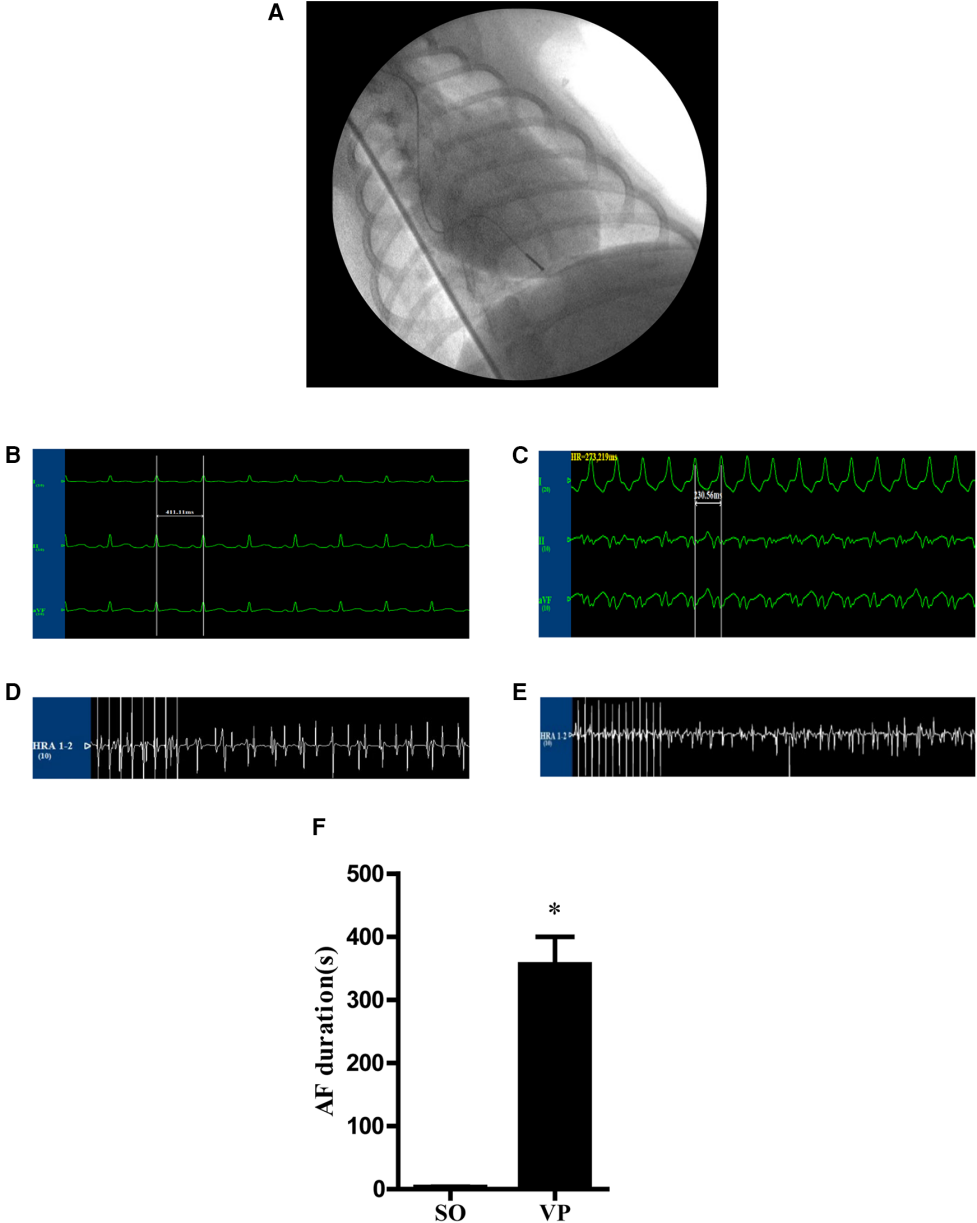
**(A)** A unipolar pacemaker lead was placed in the right ventricular apex. **(B,C)** Electrocardiograms of canines. **(B)** Potential of limb leads under the normal state. **(C)** Potential of limb leads during rapid ventricular pacing at 240–280 bpm. **(D,E)** The potential of the right atrium stimulated for 10 s at a frequency of 10 Hz and 4 times the pacing threshold to induce AF **(D)** in the SO group and **(E)** in the VP group. **(F)** The AF duration in the SO and VP groups.

For the experimental model for AF, rural dogs aged 6–7 years and weighing 10–12 kg were used both in the sham operation group (SO group) and the ventricular pacing group (VP group). The animals were anesthetized using intravenous sodium pentobarbital (20 mg/kg). Heart rate and rhythm were monitored using a continuous 3-lead electrocardiogram (LEAD-7000, multichannel physiology recorder; Sichuan Jinjiang Electronic Science and Technology Co., Ltd., Sichuan, China). A subcutaneous pocket was created anterior to the first rib to insert the pacemaker generator. In the VP group, a unipolar pacemaker lead was placed in the right ventricular apex under fluoroscopic visualization via the right external jugular vein (Figure 4A). Ventricular pacing was maintained at 240–280 bpm, and after confirmation of appropriate pacing, the incisions were closed, and the animals were allowed to recover in their cages. The animals were kept under daily observation, and a formal clinical evaluation was conducted twice weekly.

Chest roentgenograms were obtained immediately after the first surgical procedure and thereafter at weekly intervals for control purposes and to confirm the location of the lead tip. Two weeks later, the dogs were reanesthetized. Intravenous metoprolol tartrate (200 μg/kg) and atropine sulfate (40 μg/kg) were administered to block the autonomic influence on the heart, and the intravenous drip of 20 μg/(kg-h) metoprolol tartrate and 7 μg/(kg-h) atropine sulfate was maintained during the experiment. The pacemaker was removed, and after the dogs recovered sinus rhythm, right atrial stimulation was given by a programmed stimulator (DF-5A, Suzhou Dongfang Electronic Instruments Plant, Jiangsu, China). The pacing threshold was measured, and the output voltage was set at 2 times the pacing threshold. The basal stimulus (S1S1) was 300 ms in circumference, the premature beat interval (S1S2) was decreased by 2 ms, and the stimulus delay was 1 s. The longest S1S2 interval without atrial excitation was the AERP (20), which was repeated three times and averaged. The right atrium was stimulated for 10 s at a frequency of 10 Hz and 4 times the pacing threshold to induce AF three times, and the mean duration of AF was calculated for each dog. After induction of AF, the animals were sacrificed by air embolization, and the heart was quickly removed. The left atrial tissue was washed with cold saline and stored at −80°C in liquid nitrogen or stored in formalin for further analysis. Ultimately, five dogs with a mean AF duration exceeding 180 s were considered successful chronic AF models and were included in the final VP group for subsequent analysis. The SO group also comprised five dogs, subjected to the same AF induction protocol and harvested using identical methods.

### RNA extraction and quantitative real-time PCR (qRT-PCR)

TRIzol reagent (Ambion, USA) was used to extract total RNA from heart tissue based on the manufacturer's protocols. cDNA Synthesis Kit (Quanto-miR cDNA Synthesis Kit, 0960201, Beijing, China) were used to make complementary DNA from 1 μg of RNA. qRT-PCR was performed to quantify miRNAs using SYBR Green PCR Master Mixture (Quantobio, No. 0960211, Beijing, China) and an ABI Prism 7900HT sequence detection system (Applied Biosystems, Foster City, CA). The primers used for miRNA qRT-PCR were shown in Supplementary Table S1, and U6 was the miRNA assays internal control. All samples were run in triplicate, including blank controls without cDNA. The cycle threshold (Ct) was determined as the number of cycles required for the fluorescent signal to cross the threshold in qPCR. Quantification was performed using ΔCT calculation using the relative expression software tool with default settings (21).

### Masson's trichrome staining

The fibrosis of left atrial tissue was assessed by Masson's trichrome staining. Paraffin sections (3–4 μm) were dewaxed, stained with Harris hematoxylin for 3 min, rinsed with running water, differentiated with 1% hydrochloric acid alcohol for 3–5 s, washed with running water, warmed to blue for 1 min, rinsed with running water for 3 min with ponceau fuchsin, rinsed with distilled water, differentiated with 1% phosphomolybdic acid for 1 min, wiped to remove the residual phosphomolybdic acid on the slide, counterstained with 2% aniline blue for 1 min, rinsed with 95% alcohol, and dehydrated with 95% alcohol. Collagen was detected using the ImageJ threshold tool in the separated red channel. Quantification of percentage of fibrosis area stained in green related to total myocardial area.

### Caspase-3 activity assay

The left atrial tissue was homogenized to assess caspase-3 activity using a caspase-3 activity kit (Beyotime, C1116) according to the manufacturer's instructions. Fifty microliters of lysate was added and centrifuged, the supernatant was extracted, and the protein concentration was measured by the Bradford method. Another part of the protein sample was taken, 50 μl of lysate was added to the reaction, 50 μl of 2X reaction buffer (containing 10 mM DTT)and 5 μl of 4 mM DEVD-*ρ*NA (final concentration 200 Um) were added, and the water bath was maintained at 37°C for 1∼2 h. Samples were measured with an ELISA reader at an absorbance of 405 nm. The detailed analysis procedure is described in the manufacturer's protocol.

### Statistical analysis

GraphPad Prism software (GraphPad Software Version 5.01, La Jolla, CA) was used for statistical analysis. The results were compared by the Unpaired *t*-test, Two-tailed, 95% Confidence Intervals, and data are presented as the means ± standard errors. *P* < 0.05 was considered statistically significant. REST analysis was used for qPCR analysis; the formula for the expression analysis is as follows:

$$R=\frac{{\left(E_{\mathrm{target}}\right)}^{\Delta \mathrm{CPtarget}(\mathrm{MEAN}\;\mathrm{control}\text{-}\mathrm{MEAN}\;\mathrm{sample})}}{{\left(E_{\mathrm{ref}}\right)}^{\Delta \mathrm{CPref}(\mathrm{MEAN}\;\mathrm{control}\text{-}\mathrm{MEAN}\;\mathrm{sample})}}$$

## Results

### Identification of DEGs between AF and SR in heart tissues

**Figure 1:**
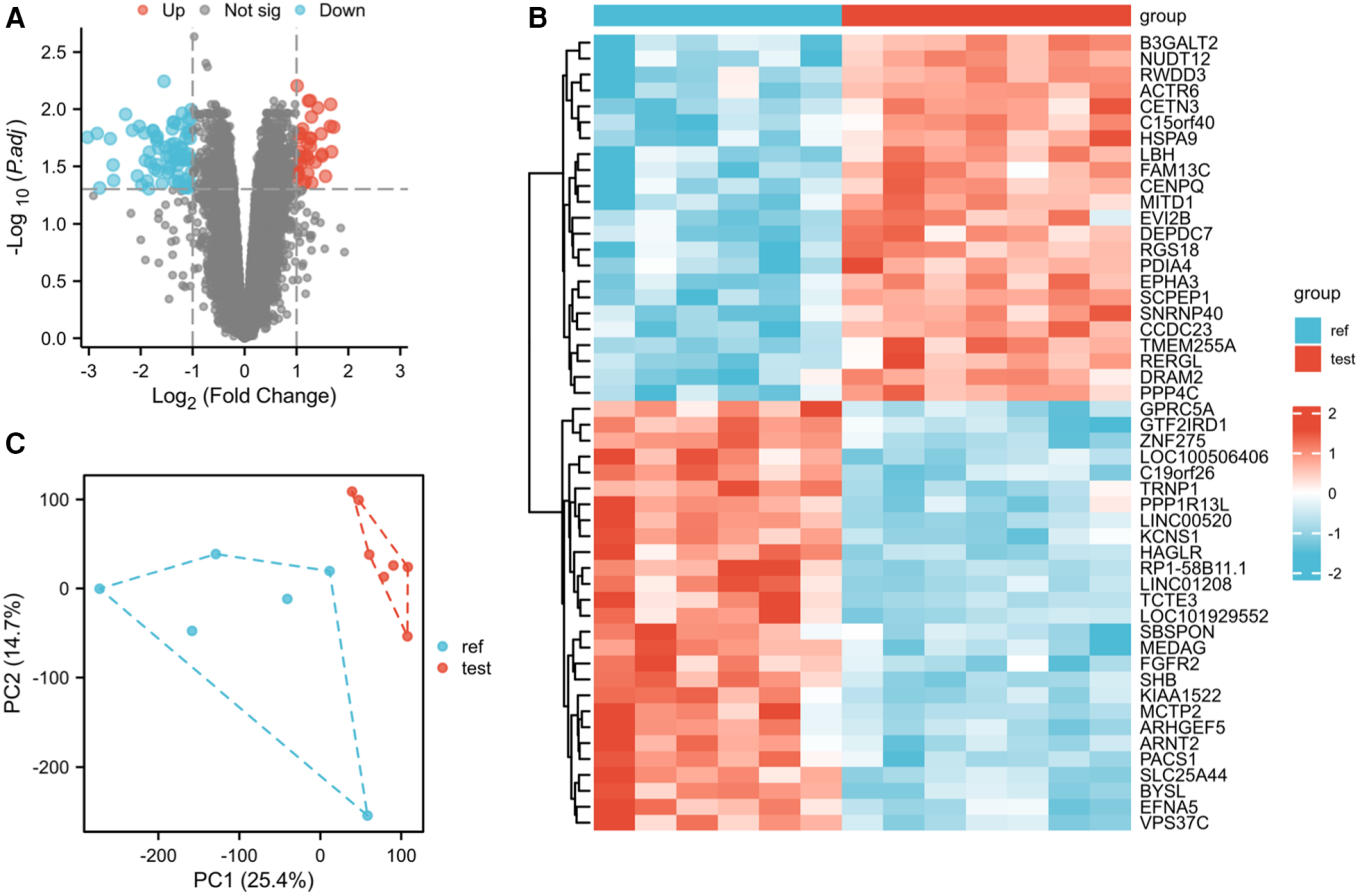
Differentially expressed gene identification. **(A)** Volcano map of GSE79768 for the LAA group. **(B)** Heatmap of GSE79768 for the LAA group. **(C)** PCA cluster plot of GSE79768 for the LAA group.

In the LAA tissues, a total of 114 DEGs were identified: 37 upregulated and 77 downregulated genes (Figure 1A). The results of mRNA differential expression analysis are presented using a heatmap and volcano plot (Figures 1A,B). The volcano plot clearly shows the distribution of upregulated and downregulated DEGs in the AF group compared to the SR group in different tissues. The heatmap displays hierarchical clustering of altered transcription in various groups. The score plots demonstrated a clear separation between the groups (Figure 1C). These analyses provided insights into the characteristics and functions of common and unique DEG transcripts.

### Protein–protein interaction network analysis

**Figure 2:**
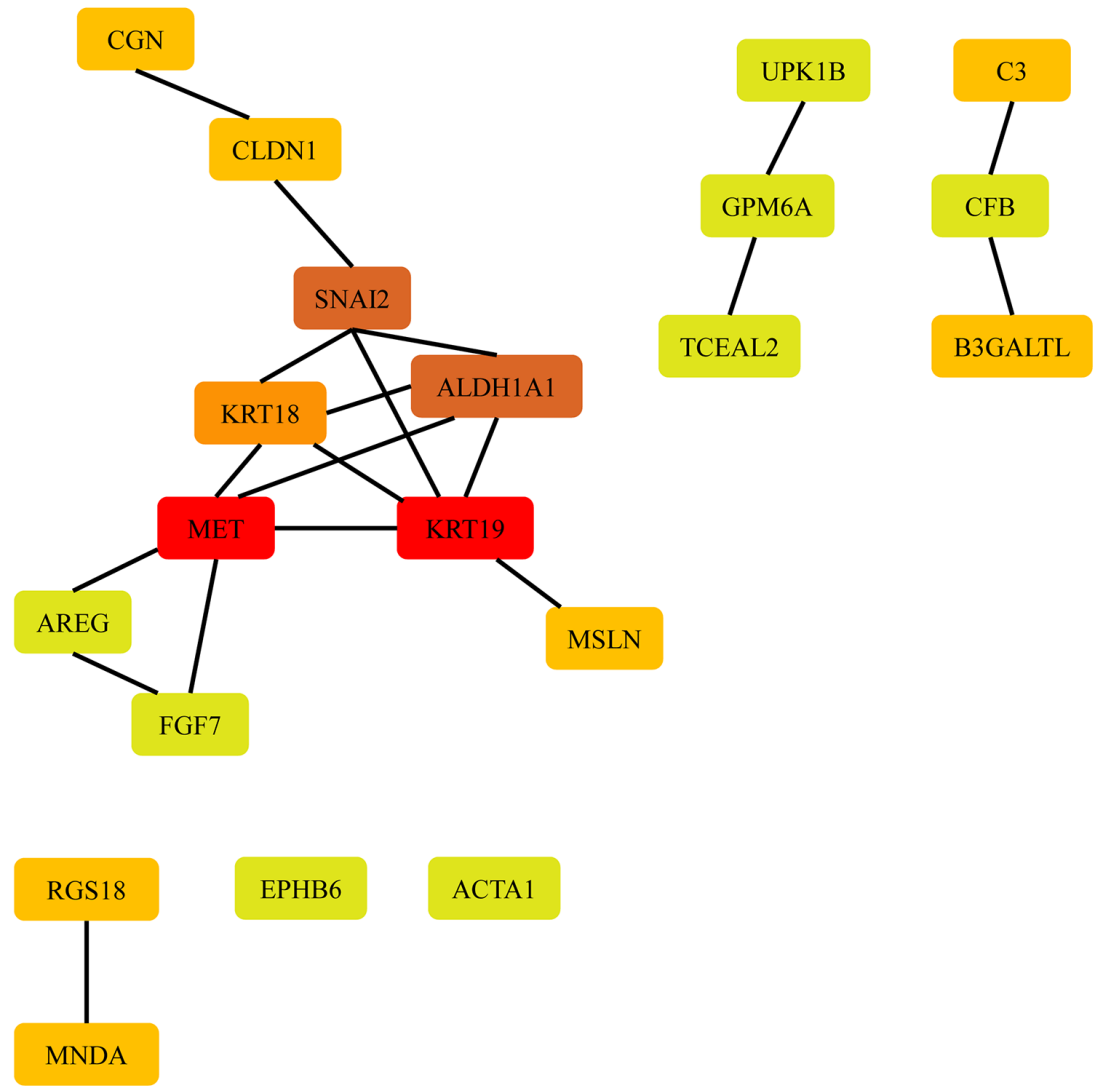
Hub gene screening. PPI network of the top 20 hub genes in LAAs.

To investigate the potential biological functions of the DEGs identified between the AF and SR groups in various tissues, we employed the STRING database to construct a PPI network. Cytoscape software was used to visualize the PPI network, and disconnected nodes were hidden. CytoHubba, a Cytoscape plugin, was used to rank the nodes in the preloaded PPI network based on various topological algorithms. In this study, we utilized the MCC method, which is known to perform better in predicting essential proteins, to identify the hub genes. The top 20 nodes in the PPI network were selected as the hub genes and are displayed in Figure 2.

In LAAs, the top 20 hub genes for upregulated differentially expressed mRNAs (DEMs) were FGF7, RGS18, MNDA, B3GALTL, ALDH1A1, and SNAI2, while for downregulated DEMs, the top 20 hub genes were CGN, CFB, CLDN1, AREG, EPHB6, ACTA1, UPK1B, GPM6A, TCEAL2, MSLN, MET, KRT19, KRT18, and C3 as presented in Table 1.

**Table 1:** The top 20 DEGs between AF and SR tissues.

Up	DEGs	log FC	adj *p*.value
	FGF7	1,655	0,014
	RGS18	1,411	0,010
	MNDA	1,359	0,029
	B3GALTL	1,002	0,031
	ALDH1A1	1,228	0,028
	SNAI2	1,142	0,026
Down	DEGs	log FC	adj *p*.value
	CGN	−1,350	0,043
	CFB	−1,978	0,046
	CLDN1	−2,063	0,038
	AREG	−1,666	0,035
	EPHB6	−1,447	0,026
	ACTA1	−1,700	0,018
	UPK1B	−3,025	0,018
	GPM6A	−1,407	0,020
	TCEAL2	−1,447	0,033
	MSLN	−2,840	0,016
	MET	−1,021	0,027
	KRT19	−2,792	0,049
	KRT18	−1,759	0,015
	C3	−1,237	0,028

### Construction of the miRNA–mRNA network

**Figure 3:**
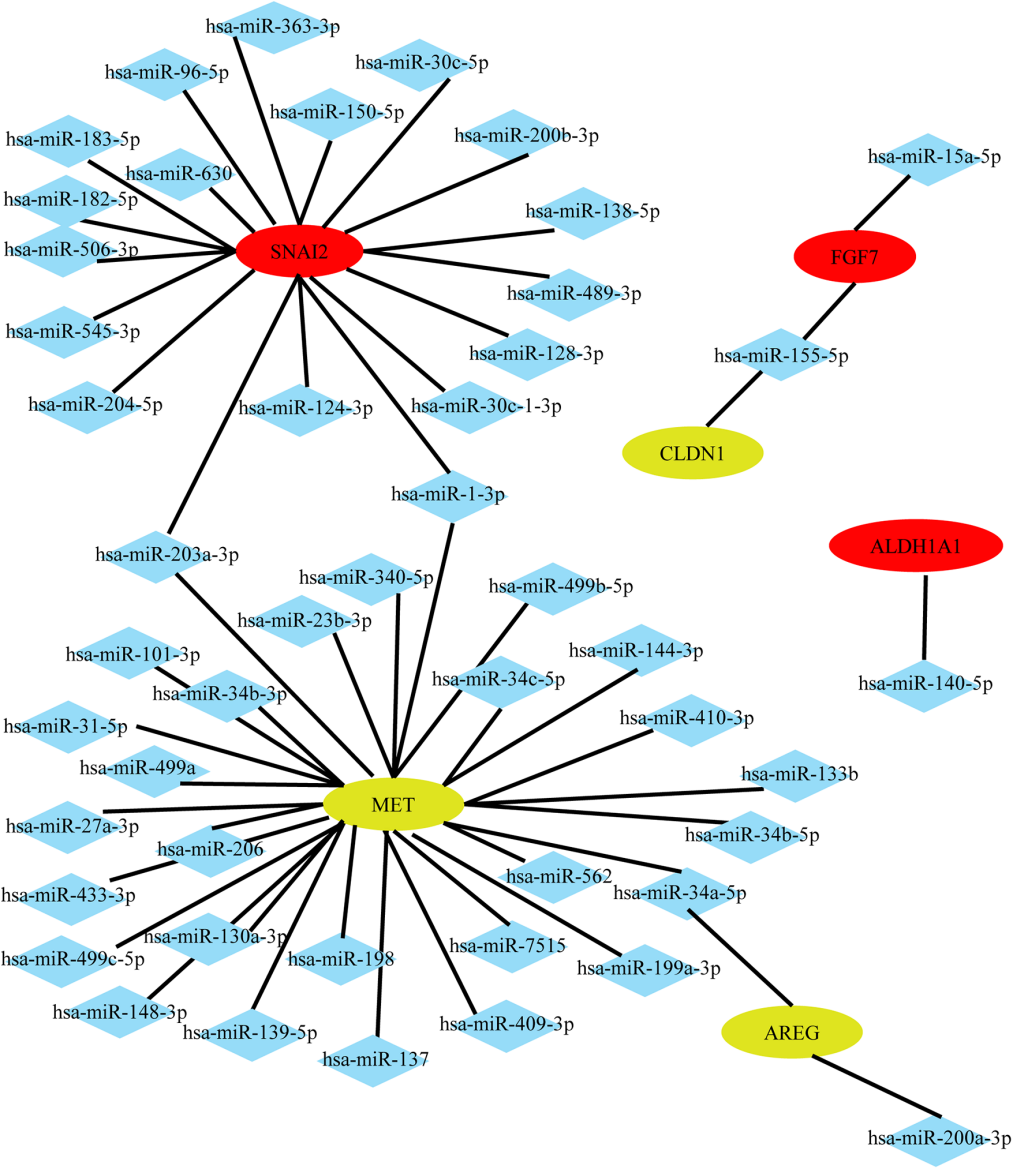
Potential miRNAs predicted by the top 20 hub genes in LAAs. Upregulated genes are colored red, and downregulated genes are colored yellow.

Identification of miRNAs associated with the top 20 hub genes of DEMs in AF and SR groups in different heart tissues was performed using the miRTarBase Web tool. Only miRNAs with strong evidence for association with mRNA were included. A network diagram was constructed using Cytoscape software, as shown in Figure 3. Notably, not all mRNAs can be associated with miRNAs. Figure 3 shows that some mRNAs can be associated with the same miRNA. For instance, in LAAs, both hsa-miR-203a-3p and hsa-miR-1-3p were associated with SNAI2 and MET; hsa-miR-155-5p was associated with both CLDN1 and FGF7.

### Right atrial potentials and ECG recordings

ECGs were obtained before pacing (Figure 4B) and after pacing (Figure 4C). Figures 4D,E shows representative right atrial potentials after 10 s of rapid atrial pacing in the SO group and the VP group. Figure 4F shows the mean AF duration time in the SO group is 3.33 ± 0.75s and 356.30 ± 43.48s in the VP group, which were statistically different.

### Effects of ventricular pacing after 2 weeks on atrial fibrosis and apoptosis

**Figure 5:**
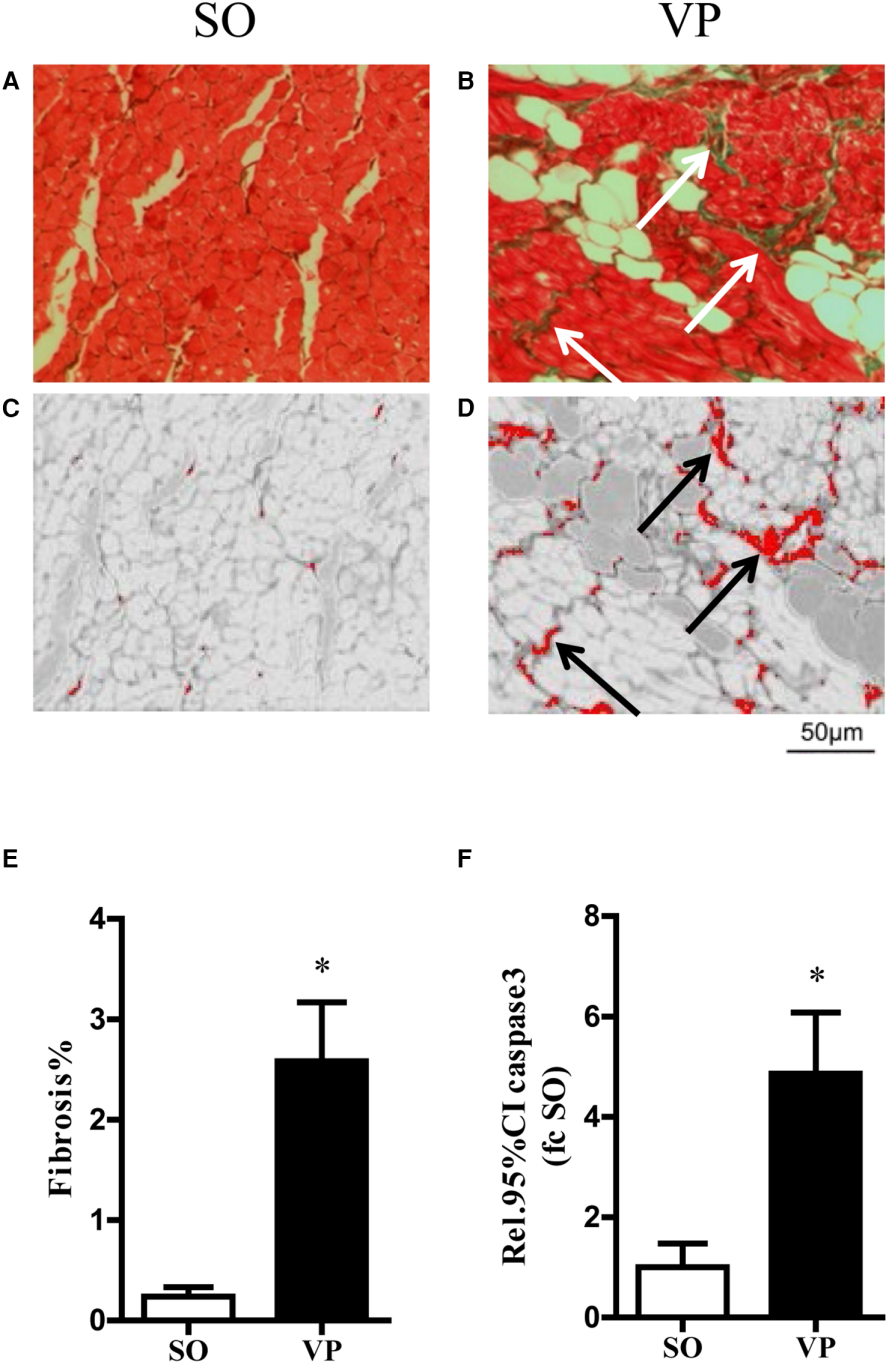
Exemplary masson trichrome stained on transversally cut histopathologic sections and fibrosis area stained in green in the SO **(A)** and VP **(B)** groups. Collagen was detected using the ImageJ threshold tool in the separated red channel **(C,D)**. Detected areas are labeled in red. **(E)** The quantification of fibrosis area related to total myocardial area in the SO and VP groups. **(F)** Caspase 3 activity of the left atrium in the SO and VP groups was analyzed by ELISAs (*n* = 5; *p < 0.05 SO vs. VP group; SEM).

The left atrial specimens were red in myocyte cytoplasm after Masson staining, and collagen fibers were stained green. Figures 5A,B show representative images of Masson staining of the left atrial tissue after 2 weeks of ventricular pacing in the SO and VP groups, respectively. Figures 5C,D shows the collagen which was detected by the ImageJ threshold tool in the SO and VP groups, respectively. The percentage of fibrosis area exhibited a significant difference between the SO and VP groups (Figure 5E). Figure 5F shows that 2 weeks after pacing, caspase 3 activity was significantly higher in the VP group than in the SO group.

### Validation of miRNA expression association

**Figure 6:**
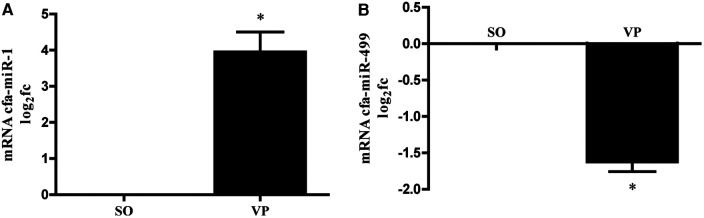
MiRNA was isolated from the left atrium followed by qPCR for miR-1 **(A)**, miR-499 **(B)**. Data are presented as the fold change (fc) compared to the SO group (*n* = 5; *p < 0.05 SO vs. VP group; SEM).

We measured the levels of miR-1, and miR-499 in left atrial tissues from the VP group. We found that the levels of miR-1 was significantly increased compared to those in the SO group, with miR-1 elevated nearly 4-fold (Figure 6A), while the level of miR-499 was significantly decreased 1.5-fold (Figure 6B).

## Discussion

AF is the most common sustained cardiac arrhythmia originating in the atrium and is associated with significant morbidity, disability, and mortality. The treatment of AF has become a critical focus in cardiovascular medicine due to its substantial global burden (22). The pathophysiology of AF is multifaceted, involving various factors such as anatomic structure, ion channels, regulatory proteins, and intercellular interactions between the conduction system, cardiomyocytes, fibroblasts, and the immune system (23). The development and maintenance of AF are believed to arise from vulnerable substrates created by electrical and structural remodeling, particularly within the left atrium (24). Therefore, to gain insights into the mechanisms underlying AF, researchers should examine the differential expression of genes in the atrium, especially in the left atrium, which can be accomplished through bioinformatics analysis.

Subsequently, target prediction and network analysis methods were used to assess PPI networks. Based on the principle roles of proteins in biological function, their interactions determine molecular and cellular mechanisms that control healthy and diseased states in organisms (25). Therefore, such networks facilitate the understanding of pathogenic (and physiologic) mechanisms that trigger the onset and progression of diseases. However, only limited interaction was found in the hub genes, and two modules with more than two nodes were discovered. One of them is the interaction of SNAI2, ALDH1A1, KRT18, MET, and KRT19; the other is the interaction of MET, AREG, and FGF7. According to the information provided by the STRING database, most of the hub genes in the networks are primarily for co-expression, with no existing experimental or biochemical data supporting interactions between them, except for KRT19 and KRT18. In comparison, these hub genes were not previously reported to be associated with AF. FGF7 and AREG are known to be associated with fibroblasts, which may give them a potential relationship with AF.

Next, we predicted target miRNAs for the top 20 hub genes using the miRTarBase Web tool, and the miRNA–mRNA network was established. This study finally selected miRNA-1 and miRNA-499 for verification in the chronic AF model. As atrial fibrosis has been recognized as a typical pathological change in chronic atrial fibrillation, miRNA-1 and miRNA-499 are reportedly associated with fibrosis formation (26, 27). These miRNAs have been associated with various cardiovascular diseases (28, 29), however, whether they have a relationship with atrial fibrosis remains not fully understood. Meanwhile, elevated levels of apoptotic processes have been observed in tissue samples from the atria of patients with atrial fibrillation and mitral disease (30). MiR-1 might have adverse effects on cardiac protection (31). Increasing evidence has shown that miR-1 promotes cardiomyocyte apoptosis and inhibits proliferation (32, 33). In our animal model of AF, a significant increase in apoptosis was also observed in atrial myocytes, which may be influenced by miR-1. The role of miR-1 in AF has been previously studied, but the results have been controversial. While some studies have reported downregulation of miR-1 in the RAAs and LAAs of patients with persistent AF (34, 35), others have reported no change in miR-1 in AF patients (36), and Feldman et al. evaluate the circulating miR-1 in pre and postsurgery of postoperative atrial fibrillation (POAF) groups in comparison with no POAF (control group) and detected lower expression of circulating miR-1 in a group of POAF in postoperative coronary artery bypass grafting (CABG) surgery, but it was not statistically significant (37). Other studies observed increased miR-1 expression in patients with AF or in an AF animal model (38, 39). The discrepancy in miR-1 expression may be due to the following reasons: (1) The expression of miRNAs may be spatially specific. (2) MiR-1 is regulated by different mRNAs. As shown by our network, two mRNAs, MET and SNAI2, regulate miR-1 simultaneously, and these two mRNAs have opposite expression patterns in the LAAs of AF patients, which may be the main reason for the controversial results of miR-1 in different studies.

The MET proto-oncogene encodes the tyrosine kinase receptor for hepatocyte growth factor, which is often overexpressed in many human cancers (40). Cell lines expressing higher levels of the MET gene and protein exhibited enhanced tumor growth, metastasis, migration, and drug resistance (41). Previous studies have shown that MET is a well-known direct target gene of miR-1 in various human cancers (41, 42). In this study, we speculate that MET may also be a target gene of miR-1 in atrial fibrillation. In our bioinformatics analysis, we found decreased expression of MET in patients with AF. In our animal model of AF, miR-1 was significantly elevated by nearly 4-fold compared to that of the control group. Specifically, the relative expression level of miR-1 showed a significant negative correlation with the expression level of the MET gene. Many studies in oncology support the notion of a negative regulatory relationship between MET and miR-1. For example, research in hepatocellular carcinoma (43), in Chordoma (44), and in Colon Cancer (45) etc. Unlike in cancer patients, we observed decreased expression of MET in patients with AF by bioinformatic analysis, whereas MET is often overexpressed in cancer patients. Currently, in AF, the known target genes of miR-1 include KCNJ2/Kir2.1, GJA1/Cx43, HCN2, Hsp60, and Hsp70 (13), the regulatory mechanism of MET and miR-1 in patients with AF remains poorly studied.

Through bioinformatic analysis, we found that miR-1 is not only associated with MET but also with SNAI2. And SNAI2 also negatively regulated the transcription of miR-1 (46). Unlike the predicted low expression of MET in patients with AF, SNAI2 is predicted to be highly expressed in AF patients. SNAI2 (also known as Slug), part of the Snail superfamily, is one of the transcription factors participating in epithelial-mesenchymal transition (EMT) (47). SNAI2 is irregularly expressed in several types of malignant cancers and plays a crucial role in cancer progression (48). Currently, it is not fully understood whether SNAI2 plays a role in AF disease.

Compared to miR-1, miR-499 lacks sufficient evidence in the current clinical and basic research on the relationship between miR-499 and AF progression. Previous studies have linked circulating miR-499 to paroxysmal AF (49, 50), suggesting its potential as a biomarker for this type of AF. However, the relationship between the miR-499 family and persistent AF, particularly in cardiac tissue, is still uncertain. Given that changes in atrial structure are closely associated with the development of AF, we investigated the expression of the mRNA in the atrium, especially in the left atrium, and the associated miRNAs. Our results indicate that hsa-miR-449a, hsa-miR-449b-5p, and hsa-miR-449c-5p were associated with mRNA in LAAs, suggesting that the expression of the miR499 family in the left atrium may be involved in the development of persistent AF. In our chronic model of AF, we found a statistically significant decrease in miR-499 in left atrial tissue compared to controls, leading us to speculate that miR-499 provides a protective effect and that miR-499 in the atria reduces fibrosis, which increases when miR-499 in the atria is reduced and eventually develops into persistent atrial fibrillation. Several articles have indicated that reduced miR-499 leads to increased fibrosis; for example, Sang et al. noted that miR-499 reduced skeletal muscle fibrosis (27). We have reported that miR-499-5p suppressed the proliferation, migration, and invasion of atrial fibroblasts and collagen synthesis (51). Further experimental validation is needed to confirm these findings, such as elevating or inhibiting miR-499 expression to observe alterations in atrial fibrosis and to elucidate the underlying mechanisms, and this molecule may be a potential therapeutic target for this condition. Meanwhile, the expression of miR-499-5p in serum in patients with AF differs from that in left atrial tissue. Wang et al. discovered that serum miR-499-5p level was significantly higher in patients with AF than in healthy controls, and miR-499-5p has the potential role as a biomarker for AF (52).

A trial registered in 2019, Atrial Fibrillation in Relationship to Sleep Quality and Plasma Biomarkers (AFISBIO)-NCT03855540, has been designed to study the levels of plasmatic biomarkers in a high-risk cohort of patients with AF and several cardiovascular co-morbidities. Interestingly, miR-1 and miR-499 are among the biomarkers enlisted in the study protocol (53). Unfortunately, no study results were posted on ClinicalTrials.gov at this moment. Currently, there is limited research on the target genes of miR-499. Liu et al. identified FOXO4 and PDCD4 as direct and functional targets of miR-499-5p. And SOX6 is verified as the primary target of miR-499-5p (54). In this study, we predicted that MET has the potential to be a target gene for miR-499 in AF, and no relevant studies have yet revealed the relationship.

Animal models are valuable tools for investigating the molecular and cellular mechanisms underlying arrhythmia, as well as more complex mechanisms at the level of the whole heart. These models also offer a means for evaluating the efficacy of therapeutic interventions (23). The sinoatrial node (SAN) is the main pacemaker in canine and human hearts (55, 56). The SAN pacemaker tissue exhibits a distinct signature of proteins and receptors that regulate SAN automaticity, ion channel currents, and cell-to-cell communication. These characteristics are predominantly similar across both species (55). The animal model of rapid ventricular pacing to AF is a classic chronic AF model in which pacing time usually ranges from one week to six weeks (57, 58). Li (57) demonstrated that pacing for 5 weeks is similar to pacing for 1 week in terms of duration of AF. It is important to note that the AF induction time for each dog is quite variable, which means not all dogs will be induced with a long AF time. Combining multiple studies and our own experimental conditions, we chose 5 dogs with an average AF duration of more than 180 s after pacing for 2 weeks for the final VP group. In the histology of the VP group, we found significant fibrosis and apoptosis in the myocardium compared to that in the SO group. This chronic AF model can reflect the pathological characteristics of persistent AF to a certain extent.

Different types of AF have distinct underlying mechanisms, and different miRNAs may be involved in these various forms of AF. This article verifies the differential expression of miR-1 and miR-499 in persistent AF. Additionally, other miRNAs, such as miR-26 (59), miR-328 (10), miR-133a (60), and miR-210-3p (61), have been suggested to play roles in AF. However, the specific mechanisms and the types of AF affected by these miRNAs are not yet fully understood and require further research. This study has some limitations. First, we got limited data on protein-protein interactions of DEGs in the hub gene, and we did not obtain information on miRNA expression trends from our bioinformatics analysis. Second, due to experimental limitations, we only selected two miRNAs for analysis in our animal experiment. Additionally, we did not verify mRNA expression trends in our animal experiment.

## Conclusion

In conclusion, this study established a miRNA–mRNA network associated with persistent AF and experimentally validated that the expression of miR-1 and miR-499 in the left atrium was associated with chronic AF. Furthermore, we observed that miR-1 and miR-499 are involved in fibrosis and apoptosis in the context of AF and thus have potential as new therapeutic targets for AF. These findings contribute to a better understanding of the changes of miRNA-1 and miR-499 in chronic AF and may aid in developing effective treatments for this condition in the future.

## Data Availability

The original contributions presented in the study are included in the article/Supplementary Material, further inquiries can be directed to the corresponding author.
