# Supplementary material for: Identification of miR-1 and miR-499 in chronic atrial fibrillation by bioinformatics analysis and experimental validation
Source: Front Cardiovasc Med. 2024 Aug 16;11:1400643. doi: 10.3389/fcvm.2024.1400643 (PMC11361948; doi:10.3389/fcvm.2024.1400643)
Supplement: Supplementary file 1 [file Table1.docx]

**Table S1**

**Table S1**. List of the primers used for miRNA quantitative real-time qPCR

| **Gene** | **Sense** |
| --- | --- |
| **cfa-miR-1** | **TGGAATGTAAAGAAGTATGTA** |
| **cfa-miR-21** | **TAGCTTATCAGACTGATGTTGA** |
| **cfa-miR-499** | **TTAAGACTTGCAGTGATGTTT** |
| **U6** | **CAAGGATGACACGCAAATTCG** |
